# The impact of HIV and high-risk behaviours on the wives of married men who have sex with men and injection drug users: implications for HIV prevention

**DOI:** 10.1186/1758-2652-13-S2-S7

**Published:** 2010-06-23

**Authors:** Sunil S Solomon, Shruti H Mehta, Amanda Latimore, Aylur K Srikrishnan, David D Celentano

**Affiliations:** 1 YR Gaitonde Centre for AIDS Research and Education, Chennai, India; 2 Johns Hopkins Bloomberg School of Public Health, Baltimore, MD, USA

## Abstract

**Background:**

HIV/AIDS in India disproportionately affects women, not by their own risks, but by those of their partners, generally their spouses. We address two marginalized populations at elevated risk of acquiring HIV: women who are married to men who also have sex with men (MSM) and wives of injection drug users (IDUs).

**Methods:**

We used a combination of focus groups (qualitative) and structured surveys (quantitative) to identify the risks that high-risk men pose to their low-risk wives and/or sexual partners. Married MSM were identified using respondent-driven recruitment in Tamil Nadu, India, and were interviewed by trainer assessors. A sample of wives of injection drug users in Chennai were recruited from men enrolled in a cohort study of the epidemiology of drug use among IDUs in Chennai, and completed a face-to-face survey. Focus groups were held with all groups of study participants, and the outcomes transcribed and analyzed for major themes on family, HIV and issues related to stigma, discrimination and disclosure.

**Results:**

Using mixed-methods research, married MSM are shown to not disclose their sexual practices to their wives, whether due to internalized homophobia, fear of stigma and discrimination, personal embarrassment or changing sexual mores. Married MSM in India largely follow the prevailing norm of marriage to the opposite sex and having a child to satisfy social pressures. Male IDUs cannot hide their drug use as easily as married MSM, but they also avoid disclosure. The majority of their wives learn of their drug-using behaviour only after they are married, making them generally helpless to protect themselves. Fear of poverty and negative influences on children were the major impacts associated with continuing drug use.

**Conclusions:**

We propose a research and prevention agenda to address the HIV risks encountered by families of high-risk men in the Indian and other low- and middle-income country contexts.

## Background

Since the first cases of AIDS were described in 1981, significant progress has been made in the prevention and management of HIV disease. New challenges have continued to emerge and solutions are not always straightforward. Injection drug use and men having sex with men remain two drivers of the HIV epidemic in the developing world, a fact that is commonly overlooked in the planning and implementation of treatment and prevention programmes [1,2].

Many of these men who have sex with men (MSM) and injecting drug users (IDUs) are married; they face unique risks and social pressures in many resource-constrained settings, which place their female sex partners and, by consequence, their children at high risk for HIV and associated co-infections. Solutions for these men and their families are far less straightforward in such settings, especially when targeted behaviours are not socially accepted and may be illegal.

India is home to ~2.3 million HIV-infected persons, the third largest group of HIV-infected individuals in the world; this reflects a population prevalence of approximately 0.3% [3]. Nearly 65% of HIV infections in India are concentrated in the western state of Maharashtra and the southern states of Andhra Pradesh, Karnataka and Tamil Nadu [4], where the epidemic has been driven by sexual transmission (85%), most of which is believed to be heterosexual [5]. However, the ability to discriminate between homosexual and heterosexual transmission in India is challenging because many MSM are married and/or bisexual, and are hesitant to self-identify as homosexual or bisexual. Injection drug use drives the HIV epidemic in the north-east, but has also been increasingly recognized in other parts.

**Figure 1:**
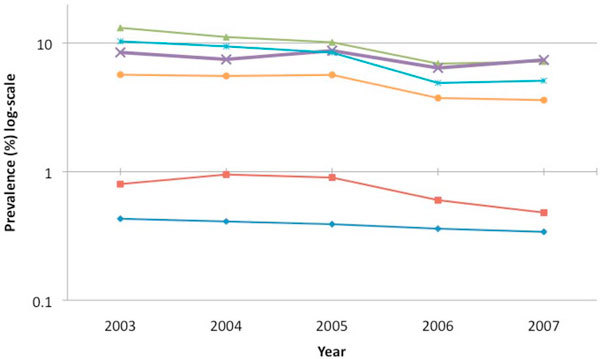
**HIV prevalence according to sentinel surveillance by risk group over time (2003-2007)**.

Recent evidence suggests that the heterosexual HIV epidemic has stabilized and may even be on the decline in the southern states (based on prevalence rates among sexually transmitted infection clinic attendees, female sex workers and women attending antenatal clinics), presumably as a result of prevention and treatment efforts and better epidemiologic assessment [6-8]. However, this declining prevalence is not reflective of all risk groups and recent sentinel surveillance data from the National AIDS Control Organization (NACO) suggest that HIV epidemics among other high-risk groups in India, such as IDUs and MSM, are not showing any signs of decline and may even be on the rise (Figure 1).

Same-sex behaviour is common in India, although overt homosexuality is rare. In a survey of male patients attending a hospital in Mangalore, Karnataka, 12%reported a sexual preference with a partner of the same sex[9]. Another sample of 2910 men from rural settings in India identified the prevalence of same-sex practices to be10% among married men and 3% among single men [5].

Cultural norms in India ensure that there are predetermined roles for women and men that impact on sexuality [10]. Women are raised from an early age to repress sexual desires and adopt the role of the obedient wife, whose primary responsibility is to reproduce. No such restrictions are placed on male children; masculinity is not defined by sexuality, but rather by fatherhood. Further, in Indian culture, close physical contact between individuals of the same gender is not considered inappropriate. Close contact between men of the same sex often begins in adolescence and, in some cases, evolves to sexual contact between men. Most men would not consider this behaviour to be inappropriate, nor would they identify themselves as "homosexual", especially when this behaviour occurs within the expectation or reality of marriage and fatherhood. Indian societal norms allow large numbers of men, who may or may not self-identify as homosexual, to have sex with men, while at the same time being married to women [10].

Although a transformation of sexual practices and awareness is certainly occurring in modern India, the open practice of a homosexual lifestyle remains uncommon. The primary reasons for this are: (1) Section 377 of the Indian Penal code, which has historically criminalized anal sex and forces many MSM to remain hidden (this law was repealed by the Delhi High Court only recently, in July 2009) [11]; and (2) the norm of marriage to the opposite sex, which results in a large proportion of MSM marrying to satisfy social pressures and/or to prove their masculinity to themselves and their families. However, a large proportion of MSM marry for the same reasons as heterosexual men - to have children, to conform with cultural norms of marriage and to avert suspicion of their sexual practices.

Epidemiologic studies have identified that between 30% and 60% of Indian men reporting same-sex behaviours are married [12,13]. Further, compared with unmarried MSM, married men tend to have higher HIV and sexually transmitted infection (STI) prevalence[14], lower rates of condom use [15,16], higher rates of anal sex, and greater numbers of sexual partners, both male and female [5].

It is likely that married MSM tend to partake in more high-risk behaviour than other MSM because of the need for anonymity. It has been reported that married MSM often indulge in hurried anonymous sex for fear of being identified as "homosexual" in social settings [17]. Despite the fact that married MSM engage in high rates of sexual risk, use of condoms with their wives is very limited. Among a sample of 821 MSM in Mumbai, India, 53% reported never using a condom with their female partners. The primary reasons for not using condoms were related to: (1) availability (33%); (2) perception that their partners were safe (32%); and (3) reduced sexual pleasure (18%) [14].

The combination of marriage to satisfy societal pressures with the observation that married MSM in particular have higher HIV prevalence and associated risk behaviour makes them an important bridge population. Their wives and children are at high risk for HIV and likely have very low risk perception. It has previously been shown in India that married women have low risk perception for HIV despite the high-risk behaviours of their husbands [18-20]. This perception is probably applicable to wives of MSM as well. Further, they remain difficult to target and reach through interventions.

India has approximately three million opiate users, the largest population in Asia [21]. Because of India's proximity to the Golden Triangle, injection drug use has been most prevalent in the north-eastern states [3]. However, injection drug use has been increasingly recognized in the southern states of India [9]. Over time, the epidemic has disseminated to other states and today, all cities with recognized injection drug use have reported HIV among IDUs, although the estimates of prevalence vary between 1% and 64% [4,8,22-26].

The majority of IDUs in India are male. Although there are limited reports of female injecting drug use in the north-east [27], most women married to IDUs are exposed to HIV through sexual contact. Given that a high proportion of IDUs (50-70%) are married, this risk is substantial. Further, IDUs put their wives and children at risk, not only because of their drug use behaviour leading to income loss, but also because they tend to have a higher than normal risk of transmitting HIV to their spouses and offspring [27-29].

Several studies have examined prevalence of HIV and STIs among sexual partners of IDUs, and have found both to be high. In a study of 332 HIV-positive IDUs from Manipur, the prevalence of HIV among spouses was 45% [30]. Another cross-sectional study among 226 IDUs and their regular sex partners in Chennai observed that the prevalence of HIV among IDUs was 30%; the prevalence among all regular sexual partners was 5%, but the prevalence was 16% among sexual partners of HIV-positive IDUs [31].

In another study examining HIV, syphilis and HSV-2 infection in IDUs and their non-injecting female partners, researchers found a 1% and 2% prevalence of syphilis in IDUs and their female regular sexual partners, respectively [32]. In addition, females with HIV-positive IDU male partners had 2.38 times the odds of having a non-HIV infection. In a convenience sample of 72 concordant and 89 discordant HIV-infected couples in Manipur, factors associated with HIV infection in wives of IDUs included current STI in either partner, as reported by the husband [33].

Despite the high prevalence of HIV and STIs among female partners of IDUs, low risk perception and low levels of HIV knowledge prevail. In a study of 3328 female regular sex partners of drug users and/or IDUs from 21 sites across India, 26.3% of women had never heard of HIV/AIDS [34]. Due to low risk perceptions, rates of condom use among these women were extremely low. In one study, female partners of IDUs with a single regular sexual partner had 40% reduced odds of condom use. A study among IDUs and their spouses in Chennai suggested that many regular sex partners viewed sex as a means of bonding, and had unprotected sex with their substance-using husbands to prove intimacy and trust in the relationship. Condoms were used only at times of menstruation or as a family-planning method, and not as a tool to protect against HIV infection [27].

This paper highlights some unique aspects of HIV epidemics among men who have sex with men (many of whom reported having sex with both men and women) and IDUs in one developing country setting, India. We illustrate some key issues regarding these marginalized populations using mixed-methods data. In particular, we highlight the impact of high-risk behaviour in these populations on female sexual partners, offer recommendations for future prevention initiatives, and identify gaps in our current knowledge of the influence of male sexual and drug use behaviours on families' risks.

## Methods

### Quantitative survey among MSM

Mixed research methods were used for both populations presented in this report. For married men with male partners, we conducted a rapid assessment to measure HIV/STI prevalence among MSM in the southern state of Tamil Nadu between October and November 2008 [35]. We recruited 721 MSM through respondent-driven recruitment, starting with 19 seeds who were identified by local non-governmental organizations as MSM, three of whom were married. We restricted our chain of referrals to three levels. Participants were eligible for participation if they: (1) were at least 18 years of age; (2) self-identified as male; (3) had a history of oral and/or anal intercourse with a man in the prior year; and (4) provided informed consent.

A structured questionnaire was administered by trained male interviewers to the identified men. The questions covered: demographics; marital history; life-time sexual history, including age at sexual debut and gender of partner, lifetime numbers of female and male partners, lifetime use of sex workers (both female and male), and other transactional sex; history of sexually transmitted diseases; recent sexual history (previous six months); and sexual concurrency. Standard laboratory assays were used to test for the presence of HIV, hepatitis C, herpes simplex virus type 2 and syphilis. We restrict the current analysis to the 247 married men who reported sex with another male.

### Quantitative survey among wives of IDUs

A similar structured questionnaire was created for the female partners of male IDUs in Chennai. A cohort study (the Madras Injection Drug User and AIDS Cohort Study) was initiated in Chennai of active IDUs (with a history of injecting in the previous six months) in 2005-06 to characterize the incidence and associated risk factors for HIV among a sample of 1158 IDUs; all but three were male [36]. From April to September 2009, we recruited a convenience sample of 400 wives and/or regular sexual partners of these men for a cross-sectional survey of their risks. Women underwent a standardized questionnaire that collected demographic information, as well as HIV risk information of both sexual and drug use practices. Women were also asked about their husbands' injection drug use patterns and the impact on their families. All women underwent testing for HIV, hepatitis C virus (HCV) and hepatitis B virus (HBV).

### Qualitative data

For married MSM, we conducted five semi-structured focus groups (each with 12 participants) in the Tamil language, led by experienced, trained facilitators. The principal targets of the groups were concerns about same-sex behaviour for the family, experiences with disclosure, how common it was to have male partners, worries and concerns about being caught having sex with a man, stigma and discrimination, consequences of coming out, and the use of alcohol and drugs prior to sex. We also inquired into reasons for and barriers to HIV testing.

We conducted similar focus group discussions with both male IDUs and their female partners in gender-specific groups in Chennai. The targets of these discussions were disclosure of injection drug use and HIV to wives, and impact of injection drug use on families of IDUs.

Research protocols were reviewed and approved by the Institutional Review Boards of the YR Gaitonde Centre for AIDS Research and Education and the Johns Hopkins Bloomberg School of Public Health.

### Statistical analysis

Quantitative data is presented primarily as descriptive with median and interquartile range (IQR) for continuous variables and number (percentage) for categorical variables. All analyses were conducted in Intercooled STATA Version 10.0 (College Station, Texas). All focus group discussions were audio-taped, transcribed into Tamil, and then coded by two individuals experienced in the analysis of qualitative data. The data were analyzed using Atlas-TI. The themes that emerged from this analysis are presented in relation to the quantitative data on infection rates and the risks that these men's behaviours pose in terms of transmitting HIV to their wives.

## Results

### Characteristics and risk behaviours of married MSM

The median age of the married MSM was 35 years (IQR, 30-42), and 75.7% had at least secondary level education. The prevalence of HIV and associated STIs among married MSM was high (HIV = 13.4%; HSV2 = 32.4%;syphilis = 11.3%). HIV prevalence among married MSM was largely explained by higher risk behaviours among married MSM, including having a greater number of male partners and not reporting a primary male partner [37].

Most (95%) married MSM self-identified as bisexual. While nearly all (97%) had disclosed their same-sex behaviour to other MSM, virtually none had disclosed their behaviour to their wives (2%), other family members (6%), and health care professionals (15%). Nearly half (51%) had been previously tested for HIV, but only 63 had received an HIV test in the prior six months, suggesting a low frequency of regular testing. Further, only four of the 33 HIV-positive married MSM were aware of their status at the time of our survey.

Half reported that they had previously received some information on HIV prevention from a counsellor. Despite this, high-risk behaviours with both men and women were common among MSM who were married. Sixty-one percent reported having a main male partner, but the majority reported having multiple male partners in the prior year (93%); 192 men (78%) reported sexual intercourse with a male commercial sex worker in the prior year; 96 (39%) reported some unprotected anal intercourse; and 26% reported always having unprotected anal intercourse with their male partners.

These married MSM also reported high-risk practices with women. Overall, 62% of married MSM had only one female partner in the prior year (wives), and 23% had multiple female partners [median: 4 (IQR: 3-8)]. One-fifth of the married MSM reported exchanging money for sex. Among those men who had sex with multiple female partners in the prior year, 88% had unprotected vaginal sex with at least one non-spousal female partner, and 128 (37%) reported vaginal sex with multiple female partners other than their wives. Three-quarters (72%) had unprotected vaginal sex with their wives in the prior year. Reported anal intercourse with spousal or non-spousal partners was rare.

### Risk context among married MSM

The qualitative data provide insight into some of the reasons for the high rates of reported risk behaviours reported by married MSM. Stigma and discrimination were identified as their biggest concerns; most participants reported fear that their families would not accept their sexuality as one of their biggest barriers to disclosure of their sexual preferences. Further, the majority concurred that the primary reasons for getting married were due to parental pressures and the fear that if they did not get married, their younger siblings would also not be able to get married, a situation that is customary in India.

Married MSM reported living in fear that their spouses would learn of their practices and divorce them. Married MSM also reported that their inability to discuss their sexuality with their children was a constant worry. In terms of substance use, smoking marijuana and alcohol use were nearly universal; the primary reason for alcohol use was personal frustration. The primary barrier to regular HIV testing was fear related to exposure of their HIV status and/or sexual practices. We also asked men in the focus groups about the high prevalence of HIV among married MSM. Men suggested that those who were married had to be more secretive about their behaviours and tended towards high-risk and multiple partnerships.

### Characteristics and risk behaviours of wives of IDUs

The median age of the women was 31 years. Thirteen percent were widowed and 7% were not currently living with their spouse; 89% reported having less than a secondary level education; and 99% reported that children were currently living in their household. Overall, risk for HIV based on their self-reported behaviours was low. Only four (1%) reported injecting drugs in the prior six months, although 22% reported non-injection drug use and 25% reported alcohol use. The majority reported only a single lifetime sexual partner (85%), and 37 (9%) reported exchanging sex for money [38].

However, risk due to their husbands' behaviours was high. Condom use was rare: 75% of the married women reported never using condoms with their husbands. As previously reported, the prevalences of HIV, HBV and HCV were 2.5%, 3.76% and 0.5%, respectively; among spouses of HIV-positive IDUs (n = 78), the prevalences of HIV, HBV and HCV were 10.3%, 1.3% and 1.3%, respectively [38].

The strongest predictor of HIV infection was spousal HIV status (OR: 17.9; p < 0.001). While all of the wives were aware of the fact that their husbands were IDUs, the majority (97%) learned of their husbands' injection practices only after marriage when they observed them injecting. The majority of the wives (84%) had seen a report of their husbands' HIV status: 68% reported that their husbands did not have HIV; 14% reported that they did have HIV; and the remainder were unsure. Risk perception in this population was actually high: nearly 60% of the women felt they were at risk of acquiring HIV, HBV and HCV from their husbands. Despite high risk perceptions, less than one-third (31%) reported that they had been tested for HIV.

We asked these women about the potential impact of their husbands' injection drug use on their family. Of 400 respondents, almost all (96.5%) were concerned that the drug use would result in the loss of income for their families and 291 (74.1%) were concerned that the drug use was a negative influence on their children. A further 218 reported that they were concerned that their husbands' injection practice placed them at high risk for domestic violence. Indeed, when we asked specifically about experiences with violence, 222 (55.5%) of the cohort reported that they were subject to some form of domestic violence, including high levels of physical and sexual violence.

### Risk context for families of IDUs

Focus groups with both the IDUs and their wives reinforced the important role of the family. The majority confirmed that women were not aware that their husbands were IDUs prior to marriage; perhaps not surprisingly, parents were often aware of their sons' behaviours. HIV-positive IDUs revealed that few spouses were aware of their HIV status; most were interested in disclosure, but needed help to do so.

We have previously reported that IDUs vacillate between living at home and on the street [39], and our focus groups confirmed that during periods when husbands are actively using drugs, wives often throw them out of the home. Further, they also confirmed the role that women might play in transitioning IDUs out of drug use. In a separate analysis from the IDU cohort, where we observed that more than 90% stopped injecting after the baseline interview, 56% and 35% reported that family encouragement and family pressure, respectively, were important in injection cessation.

## Discussion

Our data support other studies in India that have observed that a large proportion of MSM and IDUs are married. Social pressures in India lead many MSM to marry and have children despite their sexual preference for men. This forced duplicity drives many of these men underground and leads them to high-risk behaviours, putting them and their families at high risk for HIV and associated infections. Similar pressures likely drive IDUs to marry without disclosing their status to their future wives, leaving them vulnerable to HIV and associated consequences.

Not surprisingly, there are no published reports on the children of MSM or drug users, nor on the wives of MSM. Children will be challenging to study directly, as will the wives of MSM given the hidden nature of their husbands' behaviours, which drives their low risk perception. Given the differences observed in our analysis, we consider consequences and potential interventions for these groups separately.

Before interventions can be designed to reach the wives and children of high-risk men, there is a need for additional primary data from this population. However, the overwhelming challenge in obtaining such data is that women married to high-risk men are likely to be mostly unaware of their husbands' same-sex behaviour, as was demonstrated in our study. Reaching such women thus would require disclosure by their spouses of not only HIV risk and serostatus, but more importantly, of their same-sex behaviour.

Our qualitative study identified that disclosure to spouses and/or children is one of the largest burdens that MSM and IDUs face. Participants in our focus groups felt that they would face extensive levels of stigma and discrimination, not only from their immediate family, but also from the community in which they lived if they disclosed their status. Further, it is important to consider the options once disclosure takes place. Divorce, though becoming more common in India, is not the norm, especially in lower income groups.

Large-scale, community-level interventions to target stigma and discrimination towards men who are married but report same-sex behaviour may help more men disclose their status to their wives, and potentially help those who have not yet married follow a different path. The time for such interventions is ripe given the recent change in the law that no longer criminalizes anal intercourse.

However, such interventions are not without challenges. Changing community norms in a conservative culture, where religion plays a major role, will not be easy and will likely require many years of work. Open discussion of same-sex behaviour may actually backfire and result in even more stigma and discrimination targeted at MSM and their families. For these reasons, such interventions will require buy in from stakeholders (e.g., religious leaders, police force) and monitoring of ongoing community perceptions.

Another approach is to target the families of high-risk men themselves; given that the focus cannot be only on sexual behaviour, drug use or HIV, one option would be to centre these issues around access to primary health care. The idea would be that engaging families in primary health care, which carries little stigma, would open up avenues for discussions and interventions with respect to sexual health and HIV. Centres that are homosexual-friendly and offer comprehensive services (e.g., HIV testing, drug and alcohol abuse counselling) are likely to be most effective. Challenges to such interventions include sensitizing health care providers to the needs of marginalized populations to minimize stigma and discrimination, one of the primary barriers to accessing health care in our study. Care should be provided in centres that are friendly, but are not identified with any particular risk group to further minimize stigma. Finally, men should be reassured that disclosure of same-sex preference is a not a requirement of their wives receiving health care in such centres.

A major assumption made in most HIV research in India and the potential interventions described in this paper is that these women are unaware of their husbands' high-risk behaviours. However, no primary data from wives of MSM is available, and it is possible that a large number of these women may suspect or be aware of their husbands' behaviours. In such cases, interventions to provide support to these women, who are or become aware of their husbands' behaviours, are another option. Examples of such interventions include peer support groups or "hotlines" that women can call to receive anonymous support and advice.

Compared with the wives of MSM, there are more primary data available on wives of IDUs, although limited data exist on children. In some ways, interventions will be easier to implement in this population because the issues of disclosure are not as great a barrier. Our data demonstrate that health care access remains limited for the wives of IDUs and likely, by translation, for their children, too. As with MSM, interventions to provide primary health care to the wives and children of IDUs will be a first step at integrating other services, such as HIV and STI testing and counselling for domestic violence. The major barrier here is to make services affordable and accessible given the low socio-economic status of most of these families. Government centres do provide some services free of charge, but access is limited due to long waiting times. An alternate strategy would be to target increased use of the already available services. However, it would be ideal to supplement these basic services with other counselling services, such as those for domestic violence.

Interventions among high-risk populations tend to focus on the individuals themselves, including those interventions that are aimed at providing economic opportunities. India is a patriarchal society, and particularly in lower education communities, it is the husband's responsibility to earn and provide for the family while the woman tends to household activities. However, it is clear from our data that the male presence in the household is inconsistent given that these men vacillate between living at home and on the street, which negatively impacts on economic resources for most families.

While promoting stable incomes among IDUs is important, creating economic opportunities for women would both empower them and ensure a constant source of income that will enable provisions for the family when husbands cannot provide adequate income. We observed that a small proportion of these women turned to sex work to earn money for their families; alternate sources of income will prevent these women from putting themselves at even higher risk of HIV infection and will improve the quality of life for their families.

The value of family-based approaches to HIV prevention should also be recognized in other respects, both in terms of primary and secondary prevention, in addition to the provision of economic opportunity. In terms of primary prevention, optimal HIV prevention for the family is cessation of injection drug use, which will also facilitate other improved outcomes (e.g., improve economic opportunities and reduce domestic violence). Interventions to promote cessation of injection drug use do not typically involve the wives or families of IDUs. However, the nature of Indian society and the evidence from our data that family does play a key role in encouraging cessation of drug use argues for a shift from individual-focused interventions to family-focused interventions.

For HIV-positive men, secondary prevention models incorporating family-based adherence interventions for antiretroviral therapy (e.g., modified directly observed therapy) should also be extended to include wives and families to reduce further HIV transmission. Considering the current state of female-controlled prevention methods and the barriers to condom use, especially among married couples, this represents a more feasible method for women to protect themselves. Barriers to including women in such interventions include disclosure of both drug use and HIV status to the wives. However, our ability to recruit wives of IDUs into a research study and our findings from qualitative studies suggest that there is a willingness by IDUs to disclose their HIV and drug use status to their wives if given appropriate support.

## Conclusions

The Indian social and cultural context of HIV/AIDS is not dissimilar from many parts of Asia and Africa. Homosexuality and drug use are widely considered non-normative and are heavily stigmatized. Denial is rampant, and treatment for drug addiction, if available, is generally very limited or not sought. Same-sex practices and drug use are associated with social marginalization and discrimination, which is widespread. Nevertheless, available data clearly indicates that these behaviours are not rare.

The high level of bisexual concurrency among men in this study demonstrates why the Indian HIV epidemic cannot be eradicated until interventions targeted at these men and their spouses are implemented. The wives of both MSM and IDUs have little control over their spouses' risk practices, and in the case of MSM, women are probably unaware of the risks their spouses expose them to. In reality, disclosure remains the province of men, and given the stigma and discrimination perceived, it is not likely that we will see rapid increases in voluntary disclosure. The case remains much the same for wives of IDUs: while they may be far more aware of their partners' risks, there is little they can do to protect themselves from HIV.

What remains undocumented at present is the greater impact of HIV/AIDS on families: to marital stability, to household income, to food security and to the wellbeing of children. How HIV influences normal childhood development, educational attainment and prospects for future employment is unknown. In most cases, HIV leads to economic drift, which cannot have any positive features for the family.

However, these impacts on families remain speculative, with little empirical data in existence from which to draw any firm conclusions. While a rich ethnographic literature is growing [40-42], quantitative population-based evidence is not yet available. The first step in designing effective, culturally sensitive interventions will require more systematic data collection on the risks, perceptions and impacts of the husbands' high-risk behaviours in this context.

## Competing interests

The authors declare that they have no competing interests.

## Authors' contributions

SSS, SHM and DDC conceived these studies, designed the data collection methods and interpreted the data. SSS and AKS oversaw the collection of the data. SSS and SHM conducted the data analysis. All authors assisted in drafting the manuscript, and read and approved the final manuscript.
